# Adaptation and feasibility of WHO PM+ for adolescents living with HIV in KwaZulu-Natal Province, South Africa: an implementation feasibility study protocol

**DOI:** 10.1136/bmjopen-2024-088992

**Published:** 2024-07-09

**Authors:** Grace Nduku Wambua, Alan Stein, Soraya Seedat, Marit Sijbrandij, Kathy Baisley, Maryam Shahmanesh, Janet Seeley, Nothando Ngwenya

**Affiliations:** 1 Africa Health Research Institute, Durban, KwaZulu Natal, South Africa; 2 University of Oxford, Oxford, UK; 3 SAMRC/SU Genomics of Brain Disorders Research Unit, Department of Psychiatry, Stellenbosch University, Cape Town, Western Cape, South Africa; 4 Vrije Universiteit, Department of Clinical, Neuro- and Developmental Psychology, WHO Collaborating Center for Research and Dissemination of Psychological Interventions, Amsterdam Public Health Research Institute, Amsterdam, Netherlands; 5 London School of Hygiene and Tropical Medicine, London, UK; 6 University College London, London, UK; 7 University of KwaZulu-Natal, Durban, KwaZulu Natal, South Africa

**Keywords:** Adolescents, Feasibility Studies, MENTAL HEALTH, Psychosocial Intervention, PUBLIC HEALTH

## Abstract

**Abstract:**

**Introduction:**

Adolescents living with HIV (ALHIV) are an extremely vulnerable population, with the burden of mental health problems carefully documented together with the constraints for receiving timely and adequate management of the problems, especially in rural settings. Problem Management Plus (PM+) is a scalable psychological intervention for individuals impaired by distress in communities exposed to adversity. Initially developed for adult populations, few studies have assessed its potential to address adolescent distress. This study aims to co-adapt PM+ with an adherence component (PM+Adherence) for ALHIV and to evaluate its acceptability and feasibility in rural Kwa-Zulu Natal Province, South Africa.

**Methods and analysis:**

We will use a mixed-methods approach over three phases. The first phase will include a realist synthesis and collection of formative data from up to 60 ALHIV, caregivers and healthcare providers to inform the adaptation of WHO PM+, including the components of an adherence module. During the second phase, we will undertake the cultural adaptation of the PM+Adherence intervention. The third phase will involve a hybrid type 3 implementation strategy among ALHIV aged 16–19 years (n=50) to implement and evaluate the feasibility of the culturally co-adapted PM+Adherence. The feasibility indicators to be evaluated include reach, adoption, attrition, implementation and acceptability of the adapted intervention, which will be assessed qualitatively and quantitatively. In addition, we will assess preliminary effectiveness using an intention-to-treat approach on HIV-related indicators and mental health outcomes at baseline, end intervention, 2-month follow-up during the 6-month implementation.

**Discussion:**

We expect that the PM+Adherence will be acceptable and can feasibly be delivered by lay counsellors in resource-limited rural KwaZulu-Natal.

**Ethics and dissemination:**

Ethical clearance has been obtained from the University of KwaZulu-Natal Biomedical Research Ethics Committee, (BREC/00005743/2023). Dissemination plans include presentations at scientific conferences, peer-reviewed publications and community level.

**STRENGTHS AND LIMITATIONS OF THIS STUDY**

- Co-adaptation with adolescents and members of the community will provide culturally, contextually and age inclusive/appropriate adaptation of the Problem Management Plus intervention.
- This study will use existing implementation science theories, guided by the Medical Research Council framework for developing and evaluating complex interventions.
- The study is not powered to test the efficacy of the intervention; however, the sample size is sufficient for assessing feasibility, acceptability and preliminary effects.

## Introduction

 Mental health problems constitute a considerable health and disability-related burden worldwide, with young people bearing this burden,^1^,^3^ especially in middle-income countries, such as South Africa.^4^,^7^ In addition, the literature shows increased mental health problems in at-risk child and adolescent populations, such as orphanhood, exposure to violence and trauma, being ‘out of school’, socioeconomic disadvantages and high levels of deprivation.^6 7^ Another at-risk group for increased mental health problems are adolescents living with HIV (ALHIV). In addition to living with a chronic disease, ALHIV experience significant adversity related to stigma and discrimination coupled sometimes with parental illness and occasionally parental/family death, substance abuse, mental illness and living in impoverished communities.^8^,^11^ Despite antiretroviral treatment (ART) transforming HIV infection from inevitable death into a chronic condition,^12 13^ local literature highlights that the success of ART has not been without challenges, requiring daily dosing and sustained adherence to fully benefit from therapy.^14 15^ Incomplete adherence has been identified as an important driver of virological failure, drug resistance, and ultimately, progression to AIDS and death and is challenging in adolescents.^16^

ALHIV are disproportionately affected by and experience mental and behavioural health problems at higher rates than their non-HIV counterparts.^9 17^ Studies carried out in South Africa have found high rates of mental health problems, at a prevalence of 8%–12%,^18^,^20^ with increased engagement in risky behaviours among ALHIV.^8 21 22^ In addition, mental health problems have been found to adversely affect adherence to ART in this population, impacting clinical outcomes,^23 24^ with older adolescents (15–19) being the most affected.^24^ Despite the burden, the mental health needs of adolescents are often unaddressed; this is often perpetuated by low mental health awareness and literacy, limited access to mental health services as well inadequate services for adolescents and the cost of treating mental illnesses.^1 25 26^ Although an increasing number of studies have indicated a significant risk of poor behavioural outcomes that affect health and development in ALHIV, few evidence-based behavioural interventions exist to promote well-being and resilience in this population.^1721 27^,^29^ Well-being is the balance between an individual’s resource pool and the challenges they face while resilience refers to the capacity to maintain mental health despite exposure to adversity.^30 31^

A recent meta-analysis aimed to establish the differential impact of psychological interventions on mental well-being in clinical and non-clinical populations, reported that mindfulness-based and multicomponent positive psychological interventions had the greatest efficacy.^32^ They also found that positive psychological interventions, cognitive and behavioural therapy, and acceptance and commitment therapy were impactful; however, the effect sizes were moderate.^32^ The majority of the studies were from the Global North and involved general adult populations, which is not representative of the population of adolescents with HIV in South Africa.^32^ Two reviews by Bhana *et al*^21 33^ pointed out that despite the high rates of mental health problems in adolescent populations in low-middle-income countries(LMIC), there is still an urgent need to develop and evaluate evidence-based mental health treatments for ALHIV. One review^21^ examined the outcomes of interventions to support the mental health of adolescents living with or affected by HIV in LMICs. This review found seven studies conducted locally: five on HIV-affected adolescents^34^,^38^ and two on ALHIV.^11 39^ The two studies with adolescents aged 9–14 assessed the effects of a adapted 10-session family-based intervention called VUKA (‘Let’s wake up’ in isiZulu). VUKA is a culturally tailored cartoon storyline that facilitates family discussions and problem-solving to reduce risk behaviours in PHIV+youth in poverty-impacted areas.^11 39^ The intervention improved mental health, behaviour, treatment adherence and communication while stigma reduction was also noted.^11 21 39^ Another review by Bhana *et al* explored current (2014–2020) evidence-based mental health interventions for young people living with HIV or affected by HIV.^33^ Of the 13 studies identified, only 8 focused on ALHIV, with interventions varying from group-based and family-strengthening interventions to psychoeducation and disclosure. This review concluded that although there are some promising approaches, more work is needed to identify evidence-based approaches and the corresponding mechanisms of change for this population.^33^ These reviews underscore the need for brief, simple, evidence-based transdiagnostic mental health interventions for low-resource settings and task-shifting approaches owing to limited human resources.^21 33^ Despite the push to increase access to mental health counselling in South Africa, especially in primary health care,^40 41^ anecdotal findings show that adolescents rarely use health services offered on the primary care platform.^42^ Evidence suggests that at-risk adolescents globally and in South Africa underuse mental health services,^42 43^ and where youth-friendly clinics are available, they do not offer mental health support.

WHO’s Problem Management Plus (PM+)^44 45^ was identified as an appropriate intervention to address psychological distress because of its transdiagnostic and low-intensity nature. It also does not require specialists to facilitate, making it suitable for resource-limited settings. We aim to contribute to addressing this gap by culturally and contextually co-adapting PM+^44^ for use with ALHIV in rural South Africa with an additional adherence component (PM+Adherence) to address adherence concerns to ART that are common among this targeted population. In addition, we will conduct a hybrid type 3 implementation study focused on implementation outcomes to assess (1) feasibility and acceptability of PM+Adherence with ALHIV in rural South Africa and (2) evaluation of patient outcomes related to HIV and mental health.

### The intervention

PM+ was designed to be delivered face to face by trained non-specialists in five 90 min weekly sessions. The intervention takes participants through four evidence-based core strategies: stress management, problem-solving, behavioural activation and strengthening social support.^44^ The therapeutic aim of the intervention is to help deal with common mental health problems (eg, depression, anxiety and stress) and improve the management of practical problems. PM+ was developed to be facilitated individually but can also be carried out in group format.^46^ Both the individual and group versions have been found to be effective.^47^ In discussion with a local youth trial board, young people thought individual sessions would be more beneficial with group sessions to be considered after implementation if needed. Although initially developed for adult populations facing adversity, few studies assess its adaptation and utility in young people.^48 49^

### Adaptation

Despite the paucity of evidence on locally developed psychological interventions, the literature highlights the benefits of adapting interventions to promote the applicability of core principles to various contexts, thereby making them more universally available.^50^ Hall *et al* suggest that cultural and contextual adaptations are warranted when community-specific cultural contexts of risk and resilience influence disorder, and these contexts should guide efforts to design and evaluate adapted interventions.^51^ Heim *et al* highlighted the importance of adaptations made to the PM+ intervention, such as the names of the main characters and case studies, to make them locally meaningful.^52^ Additionally, they found that the adaptation of PM+ in Kenya integrated storytelling as an important feature. In Pakistan, the language was adapted to be more direct as Urdu is more direct than English (eg, phrases such as ‘we are interested in finding out’ were changed to ‘we want to know’). Culturally relevant religious practices were included in the adaptation of Pakistan and Kenya. Another adaptation in Kenya included the role of the family, in which the family was contracted to support treatment compliance.^52^

## Methodology and analysis

The first phase of this study will be a realist synthesis to identify and refine a programme theory that explains how and why WHO’s PM+ works. A realist synthesis reviews evidence to understand how, for whom and under what circumstances complex interventions function in complex environments.^53 54^ This approach was chosen because it explicitly explains how and why contexts influence outcomes and the processes/mechanisms at play.^55^ The second phase will culturally co-adapt PM+ to the local context. In the third phase, a feasibility study will examine the implementation outcomes while collecting data to evaluate patient outcomes of the culturally co-adapted PM+Adherence intervention among ALHIV.

### Study setting

KwaZulu-Natal (KZN) is one of the poorest provinces in South Africa, with high youth unemployment and HIV prevalence.^56^,^58^ Adolescents comprise approximately 11.3 million in the country, of which 2.1 million are found in KwaZulu-Natal.^59^ Although the total number of persons living with HIV (PLHIV) in South Africa increased from an estimated 3.68 million in 2002 to 8.45 million by 2022, the prevalence among the youth aged 15–24 has seen a slight decline from 6.24% in 2002 to 5.79% 2022.^59^ A community-based HIV study in KZN found that the prevalence among adolescent girls was 11.5% and 5.0% among adolescent boys aged 15–19^57^.

The study will be conducted in the uMkhanyakude (rural) district of the KZN Province, South Africa. uMkhanyakude is characterised by poverty, unemployment, poor infrastructure and temporary local migration, with family members dependent on small-scale agriculture and allowances from migrant workers and government grants. uMkhanyakude is one of the poorest districts in South Africa, with a high HIV prevalence of more than 40% and poor access to resources, with approximately 22% of the population having access to piped water and sanitation. This study is embedded within the Africa Centre Demographic Information System (ACDIS) cohort in rural KwaZulu-Natal, South Africa, established in 2000 by the Africa Health Research Institute (AHRI).^60^ AHRI is an independent, transdisciplinary scientific research institute based across two campuses in the South African province of KZN.

### Phase 1: rapid qualitative assessment

#### Study design and participants

Using the collaborative Design, Implementation, Monitoring and Evaluation approach,^61^ NN and GNW will train research staff with a minimum honours degree to conduct group free-listing interviews, in-depth interviews and participatory group discussions with ALHIV, their caregivers and healthcare professionals (HCPs). Free-listing interviews are a simple paper-and-pencil technique that makes it possible to identify, measure and describe variation in cultural knowledge between groups^62^ while establishing the coherence or boundedness of particular cultural domains to identify the items belonging to those domains and discern which items are more salient or representative of the domain.^63 64^ These will be conducted in groups which will facilitate recall and discussion on the different terminologies. We will use free lists to explore the local expressions, concepts and terminologies of mental health problems affecting ALHIV. In-depth interviews will be used to gather detailed information about the expressions, concepts and terminologies of mental health mentioned during the free-listing interviews to understand better how ALHIV conceptualise mental illness and how they navigate these mental health issues. Participatory group discussions will be built from the free lists and in-depth interviews to better understand the issues experienced by the ALHIV, help identify behavioural strategies of adherence commonly used by adolescents and explore the gender and potential age differences that might need to be considered in the intervention.

#### Sample size and sampling

In-depth and free-listing interviews will be conducted with adolescents (n=20), caregivers (n=20), HCPs (n=20) and four (n=8) participatory group discussions, two with adolescents and one with caregivers and HCPs, respectively. Participants in the free listing interviews will be invited to participate in the in-depth interviews. All participants will be purposefully chosen.

The inclusion criteria are as follows:

Adolescent participants: (1) living with HIV; (2) aged 16–19 years at the time of recruitment; (3) residence in uMkhanyakude district at the time of diagnosis and treatment; (4) who know their status; (5) in active care at one of the HIV clinics within the AHRI ACDIS and (6) willing and able to consent to participate. Potential adolescents will be excluded if they are unaware of their status and those with self-reported history of severe mental illness.

Caregivers: These will be identified by adolescent participants as primary caregivers, significant others or prominent supporters and will be recruited through nomination by the adolescent patient.

Clinician/health and social care professional participants must be nominated by adolescent participants from recruiting clinics and community-based organisations.

#### Study procedures

ALHIV will be identified from the HIV clinics in the AHRI database (ACDIS). With the help of AHRI research nurses and clinic research assistants, we will introduce the study to them and seek their consent. Adolescents’ HIV status will be checked against their clinical records. For those who consent to participate, the ALHIV will be interviewed separately from their caregiver unless the adolescent requests their presence during the interview. HCPs working with ALHIV will be recruited from participating health centres. Participatory group discussions and interviews will be conducted in a private room within the health clinics, and each group will be invited to participate on separate days. Participants in the free listing exercise will be invited to participate in the interviews.

#### Data analysis

An intervention-mapping framework and realist principles will inform the coding process that GNW and NN will lead. Thematic and narrative analyses will be conducted using a predeveloped programme theory. Part of the synthesis will involve identifying the weighting of existing subdomains and the polarity of the constructs to show how they interact and influence other health constructs. The interviews of caregivers and professionals will focus on the concepts of interest concerning the young person living with HIV, adherence and mental health. The interviews with HCPs will focus on their perspectives on adherence challenges and experiences in caring for this ALHIV population. The information gathered through these interactions will be used to develop an outcome-focused programmed theory to investigate the underlying causal mechanisms of mental health problems among ALHIV. The model will be interrogated as more data are collected to refine and clearly describe causal processes and identify any mediators of these causal influences. The gathered data will also be used to identify behavioural strategies of adherence commonly used by adolescents and to explore the gender and potential age differences that might need to be considered in the intervention.

### Phase 2: deep structure adaptions of PM+Adherence

With the findings from phase 1, we hope to understand how ALHIV make sense of their illness and how they navigate the associated challenges, including mental health and psychosocial issues. We will then use this information to adapt the intervention components purposefully.

#### Adaptation workshop

Three iterative adaptation workshops will be conducted with mentors and stakeholders (ALHIV, n=10) and healthcare providers (n=8) to review all the findings from phase one to determine recommendations for adaptation as well as the adherence components that will be incorporated into the adapted version. The identified recommendations will be compiled for suggested adaptations to intervention materials, training materials or implementation considerations. They will be coded according to the Bernal framework for cultural adaptation of psychological interventions.^65^,^67^ The Bernal framework highlights that most interventions are not generalisable to ethnic/minority populations and, thus, need contextual adaptation to reflect the broader social, economic and political contexts of intervention.^66 68^ The framework consists of eight dimensions of treatment interventions—language, persons, metaphors, content, concepts, goals, methods and context—that guide the development of culturally sensitive treatments and the adaptation of existing psychosocial treatments.^65^ During this adaptation, we will also consider implementation factors and sustainability outcomes. The final PM+Adherence intervention manual will consist of five adapted and two adherence-focused sessions with corresponding material.

#### Cognitive interviewing

A bilingual research assistant will translate the intervention material to IsiZulu, followed by cognitive interviewing. Cognitive interviewing exercises ensure the materials are easily understood, acceptable and relevant to the population.^69 70^ Following a semi-structured guide, the Youth Advisory Board, comprising ALHIV aged 16–19 years from the community, will be invited to give feedback on the adapted PM+Adherence intervention materials and give input on whether they are relevant, understandable or acceptable.^70^

#### Psychologist read-through

An expert clinical psychologist will review the adapted intervention to ensure consistency and appropriateness of the adapted intervention manual and corresponding materials. The psychologist will use an existing glossary of mental health idioms and terminology from South Africa to guide the process.

### Phase 3: feasibility trial of the co-adapted PM+Adherence

#### Study design

In this phase, we will conduct a hybrid type 3 implementation study informed by the new NIHR and UK Medical Research Council framework for complex interventions^71 72^ to determine whether implementing the co-adapted intervention is appropriate for further testing and whether the ideas and findings can be shaped as relevant and sustainable.^73^,^75^

#### Study population

We will recruit adolescents (target sample size n=50) living with HIV and receiving care at AHRI-supported clinics. A random sample of 67 potential participants (ensuring gender balance) who are aware of their HIV status and have agreed to future contact with the AHRI team will be selected from the existing AHRI Demographic Information System (ACDIS) database, and they will be approached and invited to enrol. Adolescents will be included in the study if they are aged 16–19 at the time of recruitment, reside in uMkhanyakude at the time of recruitment, have a score of ≥10 on the Patient Health Questionnaire (PHQ-9) or Generalised Anxiety Disorder Scale (GAD-7) and provide informed consent. We will exclude individuals with a self-reported history of severe mental illness, including psychotic-related disorders, bipolar disorders, eating disorders and substance dependence, as well as those with cognitive deficits (assessed using the Mini-Mental Status Examination),^76^ those presenting with suicidal ideation or judged to be at active risk of suicide (as assessed using the Columbia-Suicide Severity Rating Scale), and those undergoing active psychiatric treatment (ie, on psychotropic medication or receiving formal psychotherapy).

#### Participant timeline

Participants will be involved in the study for 6 months. Participants will complete assessments at baseline before the intervention is delivered. The intervention will be delivered over approximately 4 months, with sessions held every fortnight at the participant’s home or elsewhere. The decision to have the sessions fortnightly was due to expert feedback from local clinicians who indicated that every week would be burdensome to the young people as they need to also attend school in between clinic appointments. After completion of the intervention, participants will be scheduled for follow-up assessments at the endpoint (month 4) and 2 months post-intervention (month 6).

#### Training

Two types of training will be conducted in preparation for implementing the adapted PM+Adherence intervention. One is for research assistants (3), and another is for the intervention facilitators and lay counsellors (5). Research assistants will support the implementation of the study, including participant recruitment, collecting informed consent and outcome assessments. The research assistants will be individuals between the ages of 24 and 30 years, with a minimum bachelor’s degree, fluent in isiZulu. The research assistants’ training will cover the study’s background, methodologies, expected benefits and potential risks to participants, outcome assessment tools, administration of questionnaires and interviews, electronic data capture tools, the importance of data quality and consenting procedures. This training will be performed with the support of the AHRI Data Management team. The research assistants will also be trained on how to assess for risk, as well as monitor and record adverse and serious adverse events. For facilitators (lay counsellors) who deliver the PM+Adherence intervention, two pieces of training will be carried out in preparation for implementation. A 2-week training of trainers (ToT) workshop will be held for two trainers (both psychologists) to consider isiZulu language adaptations. The trainers will conduct supervised practice (role-play) and qualitative feedback will be collected during a debriefing session after training. A 3-month training of lay counsellors will follow ToT training. This length of time was based on previous studies using lay counsellors. The lay counsellors will be individuals between the ages of 24 and 30 years, with a minimum of matric (in South Africa, this is the grade 12 high school graduation certificate), lived experience of HIV or mental illness, from the local community with previous experience in community engagements/support. The training of lay counsellors will involve a combination of classroom and field support, followed by supervised practice in which facilitators will deliver the intervention to young people. The trainers will rate lay counsellors on fidelity and competency (ie, whether they completed each component of the session, how well they delivered the session, use of group facilitation skills, basic helping skills and how to manage acutely distressed participants). Lay counsellors will also be trained to complete self-report fidelity checklists of the intervention components. The intervention will be delivered face to face. They will also be trained to assess for risk and monitor and record adverse and serious adverse events. All research assistants and lay counsellors will be trained in safeguarding participants. The Ensuring Quality in Psychological Support platform will be consulted to select appropriate assessment tools, including WeACT–Foundational Helping Competencies for Children and Adolescents and the PM+ Competencies assessment tool.^77^

#### Study procedures

A research assistant will introduce the study, the purpose, what participation in the study involves, the length of participation, a reminder that participation is voluntary and that they can withdraw at any time without impacting their healthcare. Where possible, this will be done through a face-to-face conversation about the study; however, if this is not possible, the information will be sent via a medium (ie, email, WhatsApp) that the young person chooses and a method that will protect their privacy. Where there is a need for clarification, the research assistant will provide clarification in person or over the telephone. Potential participants will then be given time (no less than 48 hours) to look at the information sheets and ask questions about the study. The consenting process will be conducted in the participant’s native language (isiZulu) to ensure clarity and comprehension. The information sheets and consent forms will be periodically reviewed and revised as required throughout the study, such as when new safety data emerge. If changes are significant, participants will be approached for reconsenting accordingly.

##### Intervention

After informed consent, eligible individuals will be screened using the PHQ-9 and GAD-7. Individuals with a score ≥10 on the PHQ-9 or GAD-7 will be enrolled in the study and will complete a set of baseline assessments (table 1). After completing the baseline assessment, they will be assigned a lay counsellor and a date for their first interaction. Only trained lay counsellors who have undergone the proposed 3-month training will deliver the adapted PM+Adherence intervention for seven sessions held once a fortnight over 4 months.

**Table 1:** Assessment schedule

	Baseline (month 0) (t_0_)	End intervention (month 4) (t_1_)	Postintervention (month 6) (t_2_)
Demographics	X		
Viral suppression	X		X
Adherence	X	X	X
PHQ-9	X	X	X
GAD-7	X	X	X
Sleep disturbance	X	X	X
PROMIS	X	X	X
CYRM	X	X	X
PWS	X	X	X

CYRMChild and Youth Resilience MeasureGAD-7Generalised Anxiety Disorder ScalePHQ-9Patient Health QuestionnairePROMISPatient-Reported Outcomes Information System Sleep Disturbance ScalePWSPersonal Well-Being Score

##### Drop-outs

Prior to the start of the intervention, contact details of the adolescent and a significant other (caregiver/friend) will be collected. During the intervention, if a participant misses a scheduled intervention session, the research staff will contact them by phone (up to three times before they are considered lost to follow-up) to book an alternative appointment to enhance retention. Our adoption criteria is at least ≥50% of sessions attended. At the end of the intervention, to understand the reasons for participant drop-out, we will make two attempts to call or visit participants who did not complete the intervention at home.

### Study outcomes

The primary outcomes of interest include (1) reach, which consists of how many of the approached ALHIV are enrolled in the study; (2) adoption and retention, which will assess the number of enrolled participants who attend ≥50% of sessions and undergo the final assessment at 6 months; (3) acceptability of the co-adapted intervention to determine how well the target population will receive the adapted intervention, the extent to which the intervention components meet the needs of the target population and the setting and (4) implementation, which will assess fidelity to the various elements of the intervention’s protocol, including consistency of delivery as intended as well as the adolescents’ use of the intervention strategies.

#### Secondary outcomes

HIV-related outcomes include viral suppression (VL) using dried blood spots and adherence will be assessed using the Adolescent Master Protocol questionnaire, an adaptation of a self-reported adherence questionnaire that measures the number of missed doses. We will also assess the validity of these scales in our study population. Mental health and well-being outcomes will be assessed using the PHQ-9,^78 79^ GAD-7,^80 81^ Patient-Reported Outcomes Information System Sleep Disturbance Scale,^82 83^ Child and Youth Resilience Measure^84 85^ and Personal Well-Being Score.^86^

### Sample size

One of the areas of focus addressed by feasibility studies is the reach (uptake) of an intervention by the intended audience.^73^ Reach considers the absolute number, proportion and representativeness of the target population willing to participate in a given intervention and the reasons why or why not.^87^ Feasibility studies are not expected to have large sample sizes; therefore, a precision estimate was made for our sample size.^74 88^ If the true proportion of those willing to enrol (reach) is 75%, a sample size of 67 approached and 50 enrolled would allow us to estimate this proportion with a precision of ±10%. If 90% of participants complete the study at 6 months, a sample size of 50 ALHIV will allow us to estimate adoption (participant retention) with a precision of ±8%.

### Data collection and management

The data collection schedule is presented in table 1. In this phase, data collection will be conducted through repeated assessment interviews at baseline (month 0), the end of the intervention (month 4) and 2 months after the intervention ended (month 6).

The questionnaires will be self-administered and collected via a tablet computer using Research Electronic Data Capture (REDCap) tools. The forms will be checked for completeness by a research assistant soon after completion, and errors will be corrected. The REDCap database resides within a single MySQL database server within a secure server cluster at AHRI. Interviews will be audio recorded and transferred into a password-protected computer for transcription.

### Data analysis

The primary outcome of this study is the feasibility and acceptability of the delivery of PM+Adherence among ALHIV in uMkhanyakude, KwaZulu-Natal. Feasibility will be determined using the following criteria: (1) reach will be assessed through 75% recruitment and consent rates; (2) adoption and retention, where enrolled participants attend ≥50% of sessions and complete assessments at 6 months; (3) implementation, where there is at least 75% fidelity to the protocol; (4) acceptability of the intervention will be assessed through semistructured interviews (see table 2). The proportion and 95% CI for each measure will be calculated.

**Table 2:** Feasibility and acceptability measure

	Explanation	Time point	Criteria
Reach	Percentage of acceptance rate and number of adolescents recruited.	Baseline	At least 75% of the target population.
Adoption	Attendance of study participants to sessions and assessments throughout the trial.	During implementation	At least ≥50% of sessions attended.
Retention	Participants retention rates.	After implementation	A rate of 90% of participants attend the final assessment at 6 months.
Implementation	Through fidelity checklists, the research team will assess the adherence rate to the intervention protocol, including consistency of delivery as intended and the adolescents’ use of the intervention strategies.	During implementation	At least 75% or greater.
Acceptability of adapted intervention	Semistructured interviews assessing views of adolescents, facilitators and healthcare providers about the adapted intervention and implementation.	After implementation	Qualitative data will be coded for themes.

#### Quantitative data

Secondary outcomes will be assessed by comparing measurements before and after the intervention. Continuous outcomes (eg, viral load, mental health) will be analysed using paired t-tests. Logistic regression will be used to estimate the OR and 95% CIs for the association of self-reported adherence measures with VL suppression. Analysis will be conducted using STATA V.18, and statistical significance based on p<0.05.

#### Qualitative data

Transcripts from the interviews and focus group discussions will be transcribed and translated where necessary. The coding and management of data will be conducted using NVivo software.^89^ The transcripts will be coded using an inductive approach, allowing themes to emerge organically from the discussions. Their experience of the PM+Adherence intervention will be of interest, as will the identification of facilitators and barriers to engaging in the intervention and systemic related factors that provide support.

### Risk assessment and management

During screening, any potential participant identified as experiencing suicidal ideation using the Columbia Suicide Severity Rating Scale will be referred for further clinical/risk assessment by an AHRI clinical psychologist. The clinical psychologist will assess whether these are significant by considering a range of well-established factors, including history of suicidality, number of prior attempts, intention and plans. The psychologist will then decide whether they can continue with the study or require more specialised services. Although this study will not include them, we will link them with the appropriate services. Those identified with other challenges, in line with the AHRI Child Protection Standard, will be linked with the Child Protection Lead at AHRI for assessment, who will advise on further management where possible. Adverse events and serious adverse events will be closely monitored during each visit. In consultation with the Child Protection Lead, the principal investigator will determine whether these events may be related to the study and report to mentors, who will function as a monitoring board when necessary. The research assistants and facilitators will record and report these events to the study team, and serious adverse events will be reported to the ethical review committee.

### Data management and risks

The research will adhere strictly to the Good Clinical Practice (GCP) guidelines. AHRI’s database will locally store all data, prioritising patient confidentiality. Secure storage on password-protected PCs and laptops and locked drawers or filing cabinets within secure premises will be implemented for electronic and paper records. The personal details of the participants will be coded and anonymised at the source, and a unique study number will be assigned to each participant for consistent use in all study-related documentation. Only the consent form and contact details will retain personal data throughout the study. The consent forms will be scanned and saved electronically, with all paper copies destroyed after an audit process.

Participants’ names will not appear in any study documents, and identifiable data will not be transmitted outside the host Institute. Clinical data will be archived using the participant’s unique study number, ensuring separation from paper records of the consent form.

Quantitative data will undergo pseudonymisation and direct entry into the central REDCap database. The personal data of research participants will be stored on-site in password-protected files accessible exclusively to the research team. Participants will be informed about who will access their records, with details outlined in the information sheet. Data access will be limited to authorised individuals from the research team. Staff will undergo training sessions, including GCP, before the study’s initiation, and refresher training will be done per local regulations throughout the study period.

### Patient and public involvement

Active public engagement is integral to all the work we do at AHRI and this project was a result of conversations with ALHIV in a programme of work just completed investigating health literacy among ALHIV and how they negotiate life. In the programme, we worked with peer researchers who are young people living with HIV and were central to our engagement activities and advised on recruitment and retention strategies that are being used in the current project. We have worked with the Paediatric European Network for Treatment of AIDS youth advisory board in Durban, KZN, South Africa on various aspects including the development of the name for the project. As a member of the Adolescent HIV Implementation Science Alliance, we have had access to expertise and lived experience groups who advised during the development of the primary outcomes that are focused on implementation principles and strategies. Our community advisory board has also been engaged to identify some ways of introducing the study to families in the area as some young people may need support as part of their strategies in the intervention. Mental health is still quite stigmatised and so is HIV, therefore, gaining the right support within the community is quite important for us so that the young people that participate or work in this project will feel accepted in their communities. We will involve ALHIV, caregivers and HCPs in the co-adaptation of the intervention, with feedback from psychologist on the appropriateness and consistency of the adapted intervention. After the conclusion of the trial, the study team will provide participants, community advisory board, Department of Health with a summary of study outcomes.

## Ethical considerations throughout the study

Ethical clearance has been obtained from the University of KwaZulu-Natal Biomedical Research Ethics Committee, (BREC/00005743/2023), the AHRI Community Advisory Board and local Department of Health governance offices at the district and province level. Potential participants who meet the inclusion criteria will be taken through a participant information sheet describing the study’s purpose, methodology, voluntary participation and potential harm. When participants are satisfied with the contents of the information sheet and all their concerns are addressed, they will be given an opportunity to provide oral or written informed consent to participate in the study. Informed consent will be taken from participants during all phases of the study. While we expect to obtain written consent, oral consent will be considered and obtained for participants where literacy is a problem or the participant could be put at risk by existence of a paper record. Oral consent will also be obtained should other circumstances beyond the study’s control such as a pandemic as we had to during the COVID-19 pandemic due to social distancing measures and use of virtual data collection approaches. From prior work conducted at AHRI with young people, as well as engagement with our Community Advisory Board, we have received parental waiver from the ethical review committee, as some adolescents may not have disclosed their status to their parents or guardians, and we do not want to leave them out of this important research. Participants in group discussions will be advised that confidentiality cannot be guaranteed. Participants will be assured that withdrawing from the study or not being part of it will not affect the services they receive from the clinics or their patient–care relationship with the researcher. Those who decline to sign will be excluded from the study.

Participants will receive compensation following ethical guidelines and institutional reimbursement policy. Participants of group activities and in-depth interviews will receive refreshments during the session, and participants will be reimbursed for any travel expenses according to AHRI policies and an R30 (£1.50) airtime voucher as a thank-you for their time.

## Expected outcome

The burden of mental health problems facing ALHIV has been carefully documented, together with the constraints for timely and adequate management of the problems.^9 22^ Young people living in rural areas are disadvantaged by limited resources and services. We expect that through the collaboration of various stakeholders, we will successfully culturally and contextually co-adapt PM+, resulting in an appropriate intervention for ALHIV (PM+Adherence). We will also conduct a feasibility study using the adapted manual to examine implementation outcomes and minimally evaluate patient outcomes. It is hoped that the programme will improve treatment adherence while equipping adolescents with the skills to negotiate difficult situations and their general well-being. From a service delivery point of view, using lay counsellors to facilitate intervention will increase access to trained support while helping ease the burden experienced by healthcare providers.^40 50 90 91^ The effectiveness of this intervention will be assessed in a future randomised controlled trial.
