# Supplementary material for: Adaptation and feasibility of WHO PM+ for adolescents living with HIV in KwaZulu-Natal Province, South Africa: an implementation feasibility study protocol
Source: BMJ Open. 2024 Jul 9;14(7):e088992. doi: 10.1136/bmjopen-2024-088992 (PMC11243136; doi:10.1136/bmjopen-2024-088992)
Supplement: Supplementary file 1 [file bmjopen-2024-088992.reviewer_comments.pdf]

## PEER REVIEW HISTORY

BMJ Open publishes all reviews undertaken for accepted manuscripts. Reviewers are asked to complete a checklist review form (**http://bmjopen.bmj.com/site/about/resources/checklist.pdf**) and are provided with free text boxes to elaborate on their assessment. These free text comments are reproduced below.

## ARTICLE DETAILS

| | |
|---|---|
| **TITLE (PROVISIONAL)** | Adaptation and feasibility of WHO PM+ for adolescents living with HIV in KwaZulu Natal Province, South Africa: An implementation feasibility study protocol |
| **AUTHORS** | Wambua, Grace; Stein, Alan; Seeley, Janet; Ngwenya, Nothando; Shahmanesh, Maryam; Baisley, Kathy; Sijbrandij, Marit; Seedat, Soraya |

This article was not externally reviewed at BMJ Open. Protocols that have been independently assessed prior to submission to BMJ Open are usually fast-tracked to publication on the grounds that further substantial changes will not be possible. This independent assessment will usually be external, independent review for both a substantial grant award from a non-commercial or government funder and ethics approval.
